# Dynamic contrast-enhanced magnetic resonance imaging assessment of residual tumor angiogenesis after insufficient microwave ablation and donafenib adjuvant therapy

**DOI:** 10.1038/s41598-024-55416-8

**Published:** 2024-02-24

**Authors:** Ziwang Ren, Guiling Feng, Bing Li, Chuan Zhang, Yong Du

**Affiliations:** Department of Radiology, Affiliated Hospital of North Sichuan Medical College, 1 Maoyuan Road, Nanchong City, 637000 Sichuan Province China

**Keywords:** DCE-MRI, Microwave ablation, VX2, Donafenib, VEGF, Oncology, Cancer imaging

## Abstract

To analyze the correlation between dynamic contrast-enhanced magnetic resonance imaging (DCE-MRI) permeability parameters and serum vascular endothelial growth factor (VEGF) levels in a rabbit VX2 liver cancer model with insufficient microwave ablation (MWA), to observe the dynamic changes in residual tumor angiogenesis in the short term after MWA, and to assess the effectiveness of donafenib as adjuvant therapy. Forty rabbits with VX2 liver tumors were randomly divided into three groups: an insufficient MWA group (n = 15), a combined treatment group (n = 15) and a control group (n = 10). The dynamic changes in VEGF expression after MWA and the effectiveness of donafenib as adjuvant therapy were evaluated by DCE-MRI and serum VEGF levels before surgery and 1, 3, 7, and 14 days after surgery. The correlation between the volume translate constant (Ktrans) of DCE-MRI parameters and serum VEGF levels fluctuated after ablation, but the coefficient was always positive (all *p* < 0.001). Repeated-measures ANOVA revealed significant changes in the serum VEGF concentration (F = 40.905, *p* < 0.001; partial η^2^ = 0.689), Ktrans (F = 13.388, *p* < 0.001; partial η^2^ = 0.420), and tumor diameter in each group (F = 34.065, *p* < 0.001; partial η^2^ = 0.648) at all five time points. Pairwise comparisons showed that the serum VEGF level, Ktrans value and tumor diameter in the insufficient MWA group and combined treatment group were significantly lower at 1 d than in the control group, but these values gradually increased over time (all *p* < 0.05). Ktrans and tumor diameter were significantly greater in the insufficient MWA group than in the control group at 14 days (all *p* < 0.05). The serum VEGF concentration, Ktrans, and tumor diameter were significantly lower in the combined treatment group than in the other two groups at 3, 7, and 14 days (all *p* < 0.05). Ktrans is positively correlated with the serum VEGF concentration. Ktrans and the serum VEGF concentration changed significantly after treatment with insufficient ablation or in combination with donafenib, and Ktrans may change faster. Insufficient MWA promotes the progression of residual tumors. Adjuvant treatment with donafenib is effective.

## Introduction

Hepatocellular carcinoma (HCC) is one of the main causes of cancer-related death worldwide. Compared with the decline in mortality associated with all other common cancers (such as breast cancer, lung cancer and prostate cancer), the incidence of HCC-related mortality continues to increase at a rate of 2–3% per year^1^. Research on HCC treatment is characterized by multidisciplinary approach and the coexistence of multiple therapeutic approaches^2^. Ablation therapy is one of the important treatments; however, it has limited ability to remove larger or specifically located tumors and is associated with a higher risk of recurrence than surgical operations^3^. A previous meta-analysis reported that local recurrence rates in HCC patients treated with Microwave ablation (MWA) varied between 5 and 17.8%^4^. The study of residual tumors is also important due to the possibility of incomplete ablation. Close postoperative follow-up and further treatment after recurrence are essential to improve the long-term survival of patients after MWA for HCC.

Several previous studies^5–8^ suggested that incomplete thermal ablation of tumors may induce the expression of local cytokines and angiogenesis-related factors, thereby promote the growth of residual tumors; Vascular endothelial growth factor (VEGF) is highly expressed in HCC, and its signaling is the cornerstone of angiogenesis in HCC^9,10^. However, most of these methods evaluate the angiogenesis of residual tumors through pathological examination, which requires tissue specimens from residual tumors; Patients often do not undergo invasive biopsies after ablation, thus pathological examination is difficult to repeat. It can only represent the angiogenesis status at the sampling time point but not the dynamic changes in angiogenesis of residual tumors after ablation.

Dynamic contrast-enhanced magnetic resonance imaging (DCE-MRI) can provide information on blood flow perfusion in tumor tissue on the basis of lesion morphology analysis and is a noninvasive imaging method. Multiple scans can be performed at different time points on the same lesion to understand the dynamic changes in residual tumor angiogenesis. In this study, a rabbit VX2 liver cancer model with insufficient MWA was used to analyze the correlation between DCE-MRI parameters and serum VEGF levels with the aim of dynamically observing changes in residual tumor angiogenesis in the short term after insufficient MWA and evaluating the efficacy of donafenib as an adjuvant therapy. These findings will provide an experimental basis for subsequent therapeutic intervention and improving the prognosis of HCC.

## Methods

### Experimental animals and main reagents

This experiment was approved by the Committee of Animal Ethics of North Sichuan Medical College [approval number: Animal Ethics Review of North Sichuan Medical College (2023) 66]. The authors confirmed that the study is reported in accordance with ARRIVE guidelines and that all methods were performed in accordance with the relevant guidelines and regulations. The New Zealand White rabbits used for the experiments were supplied by the Centre of Experimental Animals in North Sichuan Medical College [license number: SYXK (Sichuan) 2018-076], 2.5–3.0 kg in weight, aged 4–6 months, and male only. The VX2 prototype rabbit was purchased from Hangzhou Huashu Biotechnology Co., Ltd. The rabbit VEGF enzyme-linked immunosorbent assay (ELISA) kits were purchased from Shanghai ZuoCai Biotechnology Co. Donafenib Tosilate Tablets were purchased from Zelgen Medicine Co., Ltd., Jiangsu, China.

### Experimental equipment

The magnetic resonance (MR) imaging was performed using a 3.0 T magnetic resonance tomograph (Area, Siemens, Germany) with a dedicated animal coil; the scanning parameters are given in Table 1. The contrast medium was Gd-DTPA-BMA (Omniscan) at 0.3 mmol/kg, which was injected via the ear marginal vein at 1 ml/s with a high-pressure syringe. DCE-MRI was performed with a T1 twist sequence, in which 6 phases were acquired prior to contrast injection, and then 70 phases were scanned consecutively in dynamic images, taking 4.3 s per phase.

**Table 1:** MR scanning parameters.

Scan sequence	Scanning direction	Echo time (ms)	Repeat time (ms)	Slice thickness (mm)	Slice interval (%)	Flip angle	Matrix	Field of view (mm × mm)
T1_vibe	Axial	1.29	3.26	1.8	20	12	384 × 326	206 × 300
T2_dixon	Axial	89	5970	3.0	10	120	256 × 160	278 × 330
Diffusion-weighted imaging (DWI)	Axial	84	12,800	3.0	10	90	160 × 128	244 × 310
T1_ vibe-twist_dixon	Axial	1.31	4.2	3.0	20	9	173 × 113	280 × 280

The computed tomography (CT) scanning device used was a Spiral 16-row CT apparatus (MX16, Philips, Netherland). The scan parameters were as follows: window width (WW) 250 HU, window location (WL) 35 HU, slice thickness 2.0 mm, kilovolt peak 120 kV, mAs 220, and pitch 0.984:1.

The MWA equipment used was a cooled-shaft microwave system (KY-2000, Kangyou Medical, Nanjing, China) and a 15G ablation needle antenna (KY-2450B-1, antenna length 18 cm, active tip length 1.1 cm).

The ELISA equipment was as follows: enzyme labeller (SpectraMAX Plus384 Meigu Molecular Instrument Co., Ltd.); constant-temperature water bath (HH-6 Shanghai Lichen Bangxi Instrument Technology Co., Ltd.); automatic plate washer (PW-480 Shenzhen Huisong Science and Technology Development Co., Ltd.); water purifier (SSY-II Sichuan ShuiSiYuan Environmental Protection Science and Technology Co., Ltd.); pipette gun (DaLong Xingchuang Experimental Instrument Co., Ltd.); and pipette tips (biosharp company).

### Surgical instruments

The following surgical instruments were used: 20G coaxial puncture needle (MN2020, BARD, USA); 23G needle with a 5-ml syringe, 20G needle with a 10-ml syringe, 26G intravenous indwelling needle, gauze, pressure-sensitive adhesive tape, gelatin sponge, hair removal cream, scalpel, and forceps.

### Transmission of VX2 tumors

**Figure 1:**
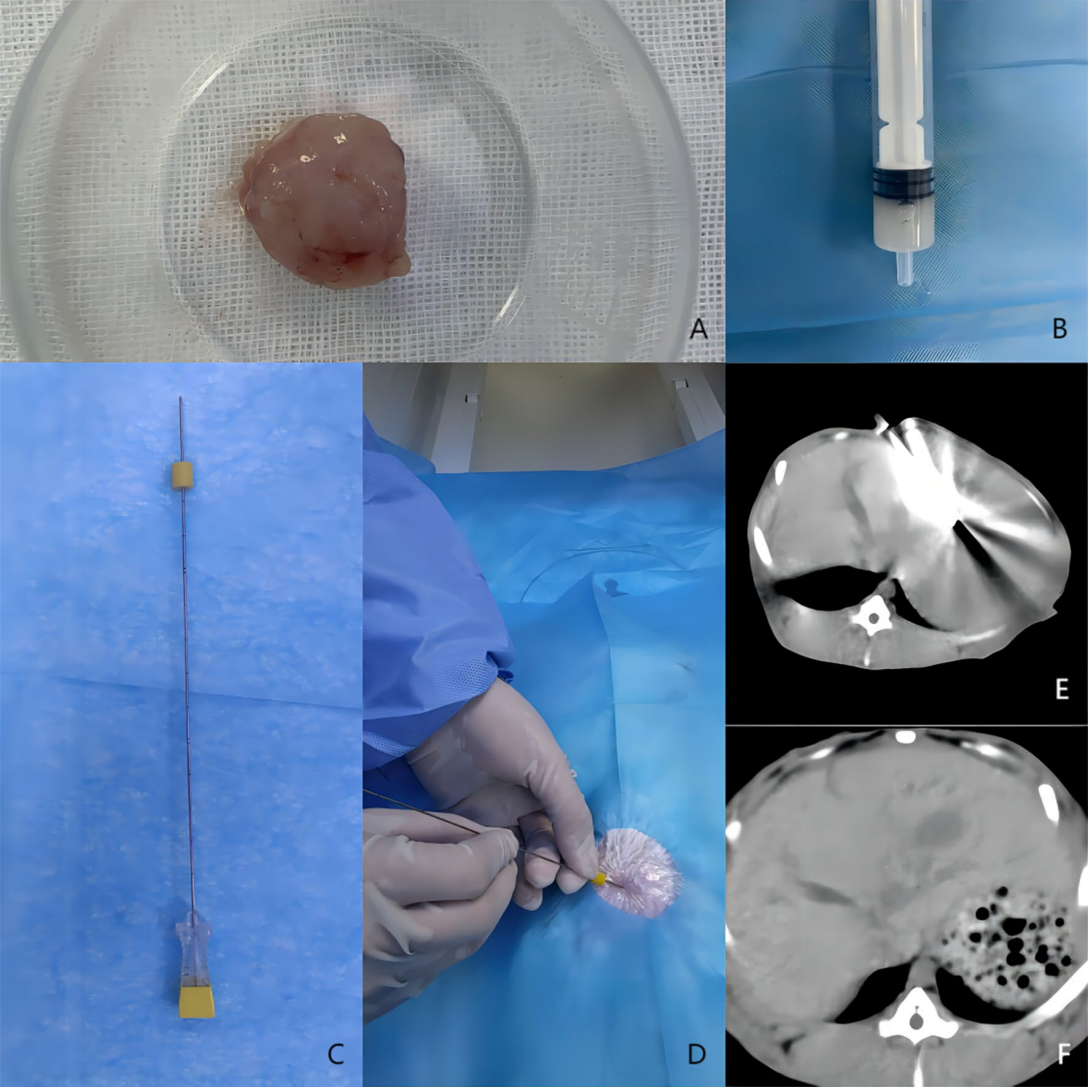
(**A**) Dissociated tumor tissue; (**B**) tumor tissue suspension prepared and loaded into a syringe; (**C**) puncture needle used; (**D**) after the skin was prepared, the puncture needle was introduced along the predetermined route; (**E**) the tip of the needle was placed in the predetermined implantation position in the liver; (**F**) CT scan before ablation, which shows the formation of a low-density lesion at the corresponding location within the liver.

After the VX2 generation rabbits were sacrificed, the posterior thigh skin was dissected to uncover the tumor tissues within the muscles and to observe the shape, size and necrosis of the lesion tissues. After stripping off the cancer mass (Fig. 1A), homogeneous fish-like tissues were removed, cut with a scalpel into tiny pieces, placed in normal saline, further crushed, milled and strained to make a suspension, which was subsequently loaded in a syringe (Fig. 1B). The posterior thigh was punctured in a normal New Zealand rabbit using a 20G coaxial puncture needle (Fig. 1C). After removing the needle stylet and aspirating with a syringe to confirm that there was no blood return, a small amount of suspension was dropped into the cannula with another syringe. The suspension was repeatedly pushed into the tissue with the needle stylet, a small amount of gelatin sponge was inserted to seal the needle channel, the puncture needle was quickly withdrawn, and local pressure was applied for 2–3 min to stop bleeding. Following the surgery, penicillin (400,000 U per day) was given intramuscularly for 3 continuous days to prevent infection. After 2–3 weeks, new VX2 tumors formed in the posterior thigh tissue, thus completing the transmission of the VX2 tumor.

### CT-guided implantation of VX2 liver tumors

After 12 h of preoperative fasting without restriction of water intake, we weighed the normal New Zealand White rabbits. An indwelling needle was inserted into the ear marginal vein of each rabbit, 3% sodium pentobarbital (1 mL/kg) was injected for general anesthesia, and the rabbits were immobilized in the supine position on the experimental operating table. Preoperative CT scans revealed no discernible anatomical variations in the livers of any of the rabbits. After skin preparation, the plants were disinfected and wiped in the subxiphoidal area (Fig. 1D), we placed a 20G coaxial puncture needle along the predetermined puncture route. The preferred location for tumor implantation was the left lobe of the liver at the level of the upper gallbladder. Once the needle tip arrived at the predetermined position (Fig. 1E), aspirated with an empty syringe, confirming that there was no blood return, and then the syringe was replaced. A small amount of the suspension (prepared using the same method as in the transmission procedure) was injected into the needle cannula and driven to the liver by repeated insertion of the needle stylet. Then, a small amount of gelatin sponge was added to the needle cannula and pressed inside the liver to seal the needle channel. The puncture needle was swiftly removed, followed by the application of local pressure for 2–3 min to stop bleeding. After ensuring that no bleeding was occurring, the surgical towel was removed, and the wound was bandaged. CT scans were performed after surgery to detect the presence of significant liver injury. Penicillin (400,000 U per day) was given intramuscularly for 3 continuous days to prevent infection. Two weeks later, CT and MR scans were obtained to monitor the size of the implanted tumor (Fig. 1F). Single intrahepatic tumors between 1.5 and 2.5 cm in diameter were successfully created in rabbits as models of liver tumors, with no apparent tumors in the remainder of the liver, no apparent metastases in the neighboring or distal tissues, and no apparent serious complications such as ascites. Forty successfully modeled hormonal rabbits were divided into 3 groups according to the random number table method (insufficiently invasive group, n = 15; combined treatment group, n = 15; control group, n = 10).

### Insufficient MWA

**Figure 2:**
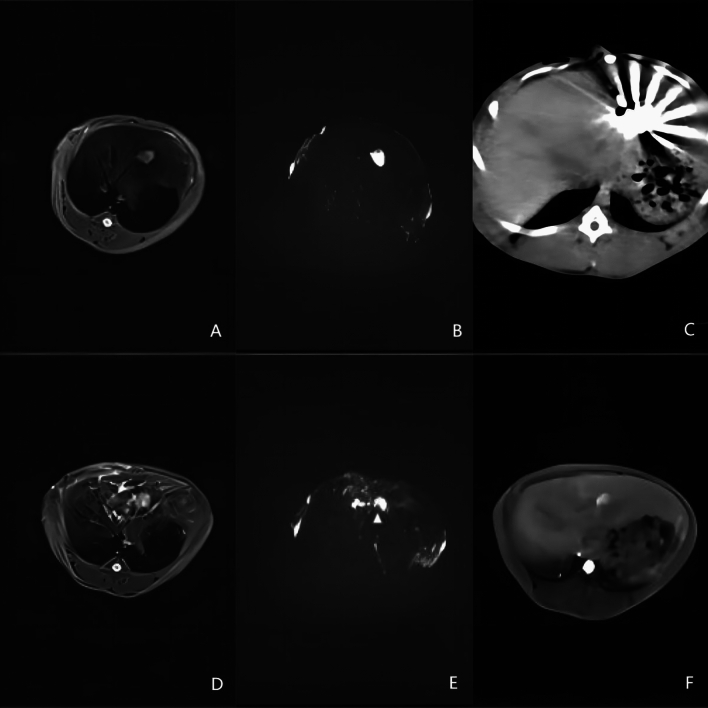
(**A**) T2WI image of the liver tumor before ablation, with the lesion showing a high signal; (**B**) DWI image before ablation, the lesion with restricted diffusion showing a high signal; (**C)** Axial CT image at the time of ablation, with the needle tip placed at the edge of the lesion and gas density visible in the ablation area; (**D**) T2WI image after ablation, with a decrease in signal in the ablated area and high signal in the residual tumor at its edge; (**E**) DWI image after ablation, the contour of the high signal area of the lesion can be seen as a defect (arrowhead), suggesting that the tumor is partially ablated; (**F**) T1WI enhancement image after ablation, residual tumor enhancement can be seen at the ablation margin.

Insufficient MWA was performed on rabbits assigned to the insufficient MWA group or the combined treatment group. After the rabbits were anesthetized, they were placed in the supine position on the surgical table. The location of the tumor was observed via MR (Fig. 2A,B) and CT before the operation; then, an experienced doctor in our department selected the appropriate percutaneous route. After preparing and disinfecting the skin at the puncture site, the ablation needle was gradually inserted following the predefined route. During CT-guided puncture, multiple scans were conducted to ensure that the needle tip followed a safe path and to minimize complications. The output power was 20–25 W, and the duration was typically 1–2 min (based on preliminary experiments, the maximum ablation diameter of a single ablation needle can reach approximately 1 cm to allow incomplete ablation) (Fig. 2C). During needle removal, heat was continuously applied for at least 5 s to prevent metastasis in the needle channel and bleeding. After the withdrawal of the needle, CT and MR scans were conducted to verify that the ablated area partially covered the tumor and that there were no serious complications (Fig. 2D–F).

### Donafenib treatment

DOLs were dissolved in normal saline to a concentration of 5 mg/mL. 15 mg/kg*d of the solution was given by gavage to each rabbit in the combined treatment group for 14 consecutive days after MWA^11^.

### Follow-up process

**Figure 3:**
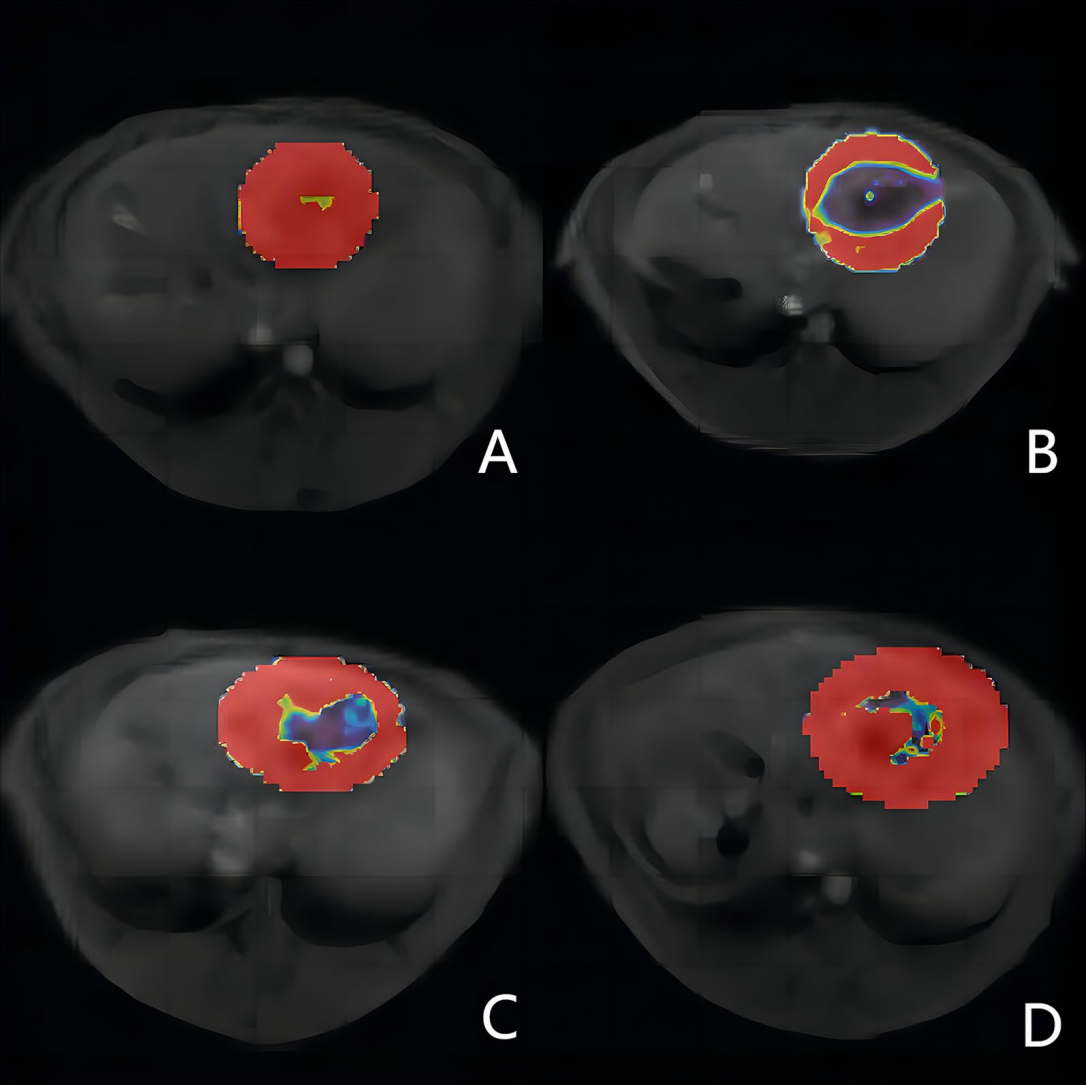
Pseudocolor maps of Ktrans of lesions in rabbits in the insufficient MWA group obtained by DCE-MRI, with colors ranging from dark blue to dark red, the shades of which indicate the values of the parameters. (**A**) Before treatment, the tumor was predominantly hyperperfused (yellow and red). (**B**) One day after insufficient MWA, perfusion in the ablated area was reduced, but hyperperfusion was still observed in the residual tissue. (**C**) Seven days after MWA, the residual tumor was enlarged, and perfusion increased. (**D**) 14 days after MWA, the area of hypoperfusion caused by ablation was markedly reduced, perfusion further increased in the ablated area, and the residual tumor increased in size.

The experimental rabbits in each group underwent DCE-MRI scans before treatment (0 days) and at 1, 3, 7, and 14 days after treatment to examine residual tumor lesions (Fig. 3), the presence of local progression, distant metastasis, and complications. In this study, the ablation range of each lesion did not exceed half of the original lesion range, and the following conditions needed to be met during subsequent follow-up MR examinations. 1. Low T1-WI and high T2-WI (slightly higher) signals can be observed in the original lesion area prior to the enhancement scan. 2. Enhanced arterial phase scanning revealed enhanced nodules in the original lesion area. 3. Limited diffusion lesions can be observed in the original lesion area. Given that we used only low power, enhancing lesions in the arterial phase and diffusion restrictions in diffusion-weighted imaging (DWI) were observed at the edge of each ablation region, and all lesions were incompletely ablated to emulate the clinical situation of insufficient ablation.

### DCE-MRI perfusion parameter extraction

Data processing was performed by Siemens Syngo. A postprocessing workstation was used. First, the images were motion calibrated to reduce the effect of respiratory motion and then combined with each sequence of images to determine the location of the tumor to place the volume of interest (VOI). The MR Tissue 4D Tofts model was selected to construct a pseudocolor map corresponding to the parameters of DCE-MRI, the solid portion of the tumor at the largest level was selected to outline the region of interest (ROI), avoiding blood vessels, calcifications, hemorrhages, and cystic degeneration areas, and the range of the ROI was 10–20 mm^2^. The DCE-MRI parameters [volume translate constant (Ktrans), reverse reflux rate constant (kep), and extravascular extracellular volume fraction (Ve)] were measured, and the tumor diameter was measured on T2W images at the same level. The positions at which each parameter was measured were kept as consistent as possible. Each parameter was measured three times, and the average value was taken as the final result.

### Serum VEGF level measurement

Blood was drawn through the middle ear artery before treatment (0 days) and at 1, 3, 7, and 14 days after treatment. The drawn blood was centrifuged at low temperature, and the supernatant was aspirated and refrigerated for subsequent ELISA analysis. All the operations were carried out in strict accordance with the operating instructions of the kit. The absorbance (OD) was measured at 450 nm using an enzyme meter, and the concentration of the sample was calculated.

### Statistical analysis

All the data were analyzed and plotted using SPSS 27.0 and GraphPad Prism 10.0. The serum VEGF levels, DCE-MRI parameters and tumor diameters at each time point were normally distributed and are expressed as the mean ± standard deviation. The correlation between each perfusion parameter of DCE-MRI and the corresponding serum VEGF levels at the same time points was assessed separately by Pearson correlation analysis. Two-way repeated-measures ANOVA was used to compare the differences in the serum VEGF concentration, DCE-MRI parameters and tumor diameter between the groups at different time points, and pairwise comparisons were made. In addition, when the data did not conform to Mauchly's test of sphericity, corrections were made using the Greenhouse–Geisser test. *P* < 0.05 indicated statistical significance.

## Results

After insufficient MWA, the correlation between each DCE-MRI parameter and the serum VEGF concentration at the corresponding time point exhibited fluctuation, except for Ktrans, which was consistently positively correlated with the serum VEGF concentration (all *P* < 0.001). The correlations between DCE-MRI parameters and serum VEGF levels are shown in Table 2. The results of the two-way repeated-measures ANOVA for the serum VEGF concentration, Ktrans, and tumor diameter are reported herein. Other DCE-MRI parameters were not reported as they did not demonstrate positive correlations with serum VEGF concentrations.

① Serum VEGF: Two-way repeated-measures ANOVA showed noncompliance with Mauchly's test of sphericity (*p* < 0.001), and Greenhouse–Geisser correction showed an interaction effect between time and group (F = 40.905, *p* < 0.001; partial η^2^ = 0.689). There was a main effect of time (F = 468.371, *p* < 0.001; partial η^2^ = 0.927). There was also a main effect of group (F = 71.925, *p* < 0.001; partial η^2^ = 0.795). Pairwise comparisons showed that there was no significant difference in serum VEGF levels between the groups before treatment. The serum VEGF concentration in the control group did not significantly change from 0 to 1 d (*p* = 1.00), but it did gradually increase over time (all *p* < 0.05); moreover, the serum VEGF concentration decreased after ablation in the insufficient MWA group and the combination treatment group compared to the preoperative level (all *p* < 0.001) but then gradually increased over time (all *p* < 0.05). The serum VEGF levels increased to similar levels in the insufficient MWA group at 3 d (*p* = 0.219) and in the combination therapy group at 7 d (*p* = 0.446), and the serum VEGF levels were similar in the insufficient MWA group and the control group at 7 d (*p* = 0.998). The serum VEGF level was greater in the control group than in the combination therapy group at 14 days (*p* < 0.001) and lower than that in the insufficient MWA group (*p* = 0.012).
② Ktrans: Two-factor repeated-measures ANOVA showed no compliance with Mauchly's test of sphericity (*p* < 0.001), and Greenhouse–Geisser correction showed an interaction effect between time and group (F = 13.388, *p* < 0.001; partial η^2^ = 0.420). There was a main effect of time (F = 93.519, *p* < 0.001; partial η^2^ = 0.717). There was a main effect of group (F = 6.140, *p* = 0.005; partial η^2^ = 0.249). The results of pairwise comparisons showed that Ktrans values significantly reduced from preoperative levels in both the insufficient MWA group and the combination treatment group after ablation (all *p* < 0.001), after which they increased in all groups over time (all *p* < 0.05). Ktrans values were similar in the insufficient MWA group and the combined treatment group at 1d (*p* = 0.731), and Ktrans values were similar in the insufficient MWA group and the control group at 3d (*p* = 0.113).
③ Diameter: Two-way repeated-measures ANOVA showed noncompliance with Mauchly's test of sphericity (*p* < 0.001), and Greenhouse–Geisser correction showed an interaction effect between time and group (F = 34.065, *p* < 0.001; partial η^2^ = 0.648). There was a main effect of time (F = 166.488, *p* < 0.001; partial η^2^ = 0.818). There was also a main effect of group (F = 15.172, *p* < 0.001; partial η^2^ = 0.451). The results of pairwise comparisons showed that the tumor diameter increased over time in the control group (all *p* < 0.05); although the ablation performed in the insufficient MWA and combination therapy groups reduced the tumor diameter (all *p* < 0.001), it still subsequently increased over time (all *p* < 0.05); the tumor diameter in the insufficient MWA group exceeded that of the control group at 14 days (*p* = 0.011), whereas the tumor diameter in the combination therapy group was lower than that of the control group at 14 days (*p* < 0.001).

**Table 2:** Correlations between DCE-MRI parameters and serum VEGF levels.

Variables	0d	1d	3d	7d	14d
Ktrans (min^−1^)	0.678 (*P* < 0.001)	0.565 (*P* < 0.001)	0.603 (*P* < 0.001)	0.664 (*P* < 0.001)	0.668 (*P* < 0.001)
Kep (min^−1^)	0.262 (*P* = 0.102)	0.296 (*P* = 0.064)	0.243 (*P* = 0.130)	0.264 (*P* = 0.100)	0.284 (*P* = 0.076)
Ve (%)	0.220 (*P* = 0.172)	0.175 (*P* = 0.280)	0.036 (*P* = 0.826)	0.305 (*P* = 0.056)	0.252 (*P* = 0.117)

The tumor diameters, perfusion parameters and serum VEGF levels in each group at different measurement times are summarized in Table 3.

**Table 3:** Variables at different measurement times.

	Variables	Control group (n = 10)	MWA group (n = 15)	Combination group (n = 15)
0d	Diameter (cm)	1.671 ± 0.142	1.678 ± 0.144	1.701 ± 0.170
	VEGF (pg/ml)	108.334 ± 4.700	112.341 ± 8.284	110.504 ± 6.773
	Ktrans (min^−1^)	0.328 ± 0.024	0.349 ± 0.039	0.343 ± 0.025
	Kep (min^−1^)	1.352 ± 0.142	1.446 ± 0.218	1.471 ± 0.204
	Ve (%)	0.245 ± 0.017	0.245 ± 0.032	0.238 ± 0.032
1d	Diameter (cm)	1.674 ± 0.142	1.307 ± 0.130	1.317 ± 0.199
	VEGF (pg/ml)	109.135 ± 3.108	74.168 ± 9.463	70.027 ± 6.377
	Ktrans (min^−1^)	0.334 ± 0.018	0.267 ± 0.057	0.273 ± 0.049
	Kep (min^−1^)	1.423 ± 0.078	1.140 ± 0.245	1.242 ± 0.165
	Ve (%)	0.249 ± 0.043	0.245 ± 0.073	0.237 ± 0.079
3d	Diameter (cm)	1.783 ± 0.135	1.591 ± 0.116	1.429 ± 0.203
	VEGF (pg/ml)	123.236 ± 5.055	115.786 ± 8.797	85.848 ± 10.521
	Ktrans (min^−1^)	0.356 ± 0.016	0.330 ± 0.044	0.304 ± 0.042
	Kep (min^−1^)	1.520 ± 0.154	1.446 ± 0.222	1.343 ± 0.157
	Ve (%)	0.237 ± 0.024	0.231 ± 0.038	0.250 ± 0.081
7d	Diameter (cm)	1.935 ± 0.126	1.897 ± 0.237	1.532 ± 0.198
	VEGF (pg/ml)	152.413 ± 9.782	152.423 ± 10.143	107.812 ± 16.547
	Ktrans (min^−1^)	0.375 ± 0.012	0.369 ± 0.046	0.323 ± 0.041
	Kep (min^−1^)	1.628 ± 0.193	1.572 ± 0.191	1.546 ± 0.152
	Ve (%)	0.234 ± 0.027	0.241 ± 0.054	0.212 ± 0.035
14d	Diameter (cm)	2.185 ± 0.145	2.473 ± 0.354	1.683 ± 0.214
	VEGF (pg/ml)	171.567 ± 9.386	188.416 ± 16.175	130.412 ± 18.238
	Ktrans (min^−1^)	0.402 ± 0.026	0.439 ± 0.035	0.355 ± 0.039
	Kep (min^−1^)	1.908 ± 0.279	1.971 ± 0.355	1.677 ± 0.217
	Ve (%)	0.215 ± 0.301	0.230 ± 0.047	0.217 ± 0.043

## Discussion

Local ablation has become the first-line treatment for patients with small HCC or patients who cannot undergo surgery; this approach can kill tumor tissue and significantly reduce tumor burden, as it is associated with minimal damage, few side effects and a short recovery time. However, the tumor recurrence rate after thermal ablation is still high. Hypoxia and hypoxia-driven angiogenesis play important roles in tumor progression, and the hypoxic environment generated by local ablation may induce the overexpression of a variety of angiogenesis-related cytokines^12,13^, among which VEGF is a key factor^14,15^. It has been well documented^16–18^ that VEGF is closely associated with the development and prognosis of malignant tumors such as HCC. VEGF and its receptor are significantly overexpressed in HCC tissues compared with normal liver tissues and are related to the high malignancy and poor prognosis of HCC^19^.

Xu et al.^20^ showed that HIF-A and VEGF overexpression after radiofrequency ablation (RFA) promoted rapid growth of residual tumors, and sorafenib inhibited residual tumor progression. However, the study was conducted using subcutaneous implantation of nude mice, which differs significantly from the blood supply of the prototype HCC tumor. Kong et al.^21^ reported that sorafenib is a feasible adjuvant therapy after insufficient MWA. But all of these studies detected VEGF expression via immunohistochemical examination of tissue samples taken after surgery; this finding could not reflect the dynamic changes in VEGF expression after MWA-induced insufficiency in the same lesion. In the clinic, patients with HCC generally do not undergo invasive examination after receiving ablative therapy, and tissue samples are difficult to obtain. Therefore, we used ELISA to detect VEGF levels in serum in this study. On the one hand, it is easy to use this material; on the other hand, ELISA is reproducible and easy to use, and it can also reflect changes in VEGF levels in real time to match each parameter obtained from DCE-MRI. Morphological evaluation alone may not be able to accurately reflect the sensitivity of the tumor to drug therapy in a timely manner, while metabolic parameters such as blood flow and metastatic parameters are associated with perfusion information inside the tumor, which may change before the size of the tumor changes. DCE-MRI can observe and evaluate the functional state of microcirculation in tissues and organs at the microvascular level^22–24^. In this experiment, we focused on the angiogenic status of the residual tumor and did not perform "sufficient microwave ablation" because the area of the completely ablated lesion contained only necrotic tissue resulting from the microwave ablation, and the ROI within the ablation area provided very few parameters.

Donafenib, which was developed by replacing the methyl group on the sorafenib molecule with a tri-deuterated methyl group, potentially enhances molecular stability, improves pharmacokinetic profiles, and has anticancer activity similar to that of sorafenib^25^. In the multicenter, randomized controlled phase II-III trial ZGDH3 conducted in China^26^, donafenib was superior to sorafenib in terms of overall survival and had good safety and tolerability profiles. Several studies^27–29^ have shown that donafenib is efficacious and very cost-effective for the treatment of HCC. However, few studies have evaluated the effect of combination therapy on residual tumors, and to our knowledge, there are no reports examining the use of MWA in combination with donafenib for the treatment of liver tumors.

In this animal study, the correlation coefficient between Ktrans and serum VEGF decreased after ablation and then gradually recovered to similar preoperative levels, possibly because the serum VEGF concentration represents the average level of systemic angiogenesis, whereas the Ktrans obtained by DCE-MRI represents the level of local angiogenesis. Specifically, although the serum VEGF concentration was significantly reduced due to the reduction in tumor volume induced by ablation, there was still abundant and dense tumor vasculature in the local residual tumors; therefore, the correlation decreased and gradually recovered over time (Table 2).

**Figure 4:**
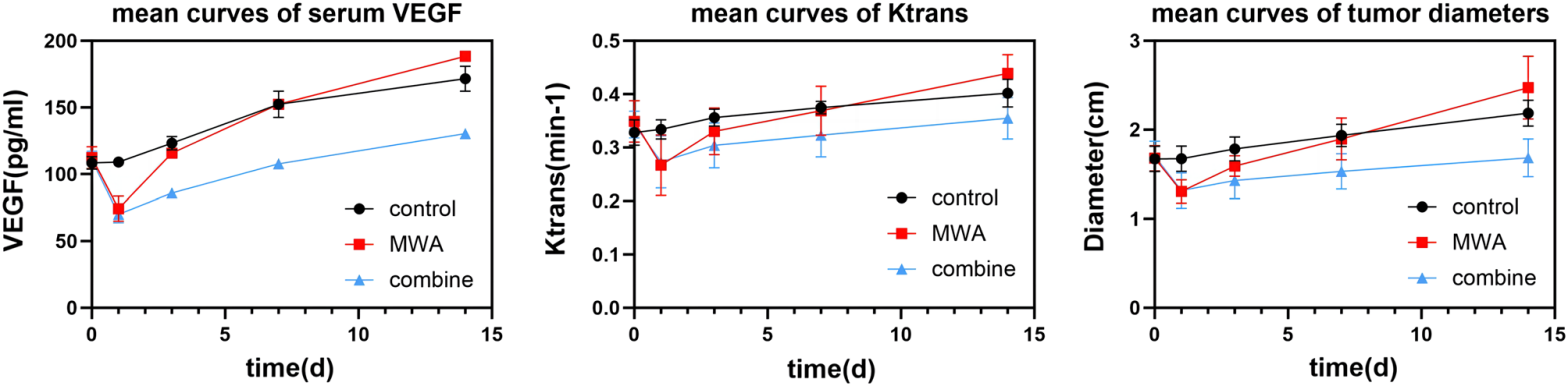
Mean curves of serum VEGF and Ktrans and tumor diameter.

The mean curves of the serum VEGF concentration, Ktrans value and tumor diameter are shown in Fig. 4. There was no significant difference in the serum VEGF levels or Ktrans values or tumor diameters between the MWA group and the combination group at 1 d after ablation (*P* = 0.123, *P* = 0.731 and *P* = 0.867), and the values were lower in both groups than in the control group (all *P* < 0.001), which may be attributed to the fact that the main effect at this time was the reduction in tumor volume. However, serum VEGF levels rose relatively quickly in MWA group, slightly below control levels at 3d (*P* = 0.045), and then rose to the same level as control group at 7 d (*P* = 0.998), the tumor diameters rose to the same level too at 7 d (*P* = 0.640), both of them in MWA group were higher than control group at 14d (all *P* < 0.05), which was consistent with previous studies suggesting that incomplete thermal ablation of tumors may promote residual tumor growth. The mean curves for Ktrans followed a similar path as the curves for serum VEGF, but Ktrans in the MWA group rose to the same level as that in the control group at 3 days (*P* = 0.113), which was quicker than that in the VEGF 7 days, suggesting that Ktrans may have a faster response rate than serum VEGF, but this needs to be verified by further experiments. Ktrans and serum VEGF levels and tumor diameters in the combined treatment group were always lower than those in the other two groups at 3, 7, and 14 days (all *P* < 0.05), suggesting that donafenib can effectively inhibit angiogenesis in residual tumors. Ktrans and serum VEGF levels and tumor diameters increased fastest in the MWA group during the 1–3 day period (Fig. 4), suggesting that antiangiogenic therapy immediately after ablation may be an appropriate time point.

There are several limitations in this study. 1. Although each experimental rabbit was assessed at multiple time points, the overall sample size of this study was still small, which may have led to some statistically nonsignificant differences. 2. The large amount of data, the cumbersome parameter measurements, and the accuracy and comparability of quantitative parameters may have been affected by the use of different MRI scanners, different DCE-MRI scanning protocols and different scanning sequences in different experiments. 3. The postprocessing workstations and hemodynamic models used in different experiments are also different, and the results obtained may be different.

## Conclusion

Ktrans is positively correlated with the serum VEGF concentration; Ktrans and the serum VEGF concentration change after treatment with insufficient ablation or in combination with Donafenib. Our findings are consistent with the findings of several previous studies showing that incomplete thermal ablation promotes residual tumor growth. Ktrans may have a faster response rate than does the serum VEGF concentration, but this needs to be verified by further experiments. Antiangiogenic therapy immediately after ablation may be an appropriate. Furthermore, adjuvant treatment with donafenib is an effective method.

## Data Availability

The data that support the findings of this study are available upon request from the corresponding author.

## Abbreviations

DCE-MRI: Dynamic contrast-enhanced magnetic resonance imaging
VEGF: Vascular endothelial growth factor
HCC: Hepatocellular carcinoma
MWA: Microwave ablation
ELISA: Enzyme-linked immunosorbent assay
MR: Magnetic resonance
CT: Computed tomography
WW: Window width
WL: Window location
VOI: Volume of interest
ROI: Region of interest
Ktrans: Volume translate constant
kep: Reverse reflux rate constant
Ve: Extravascular extracellular volume fraction
OD: Absorbance
RFA: Radiofrequency ablation
